# kngMap: Sensitive and Fast Mapping Algorithm for Noisy Long Reads Based on the *K*-Mer Neighborhood Graph

**DOI:** 10.3389/fgene.2022.890651

**Published:** 2022-05-05

**Authors:** Ze-Gang Wei, Xing-Guo Fan, Hao Zhang, Xiao-Dan Zhang, Fei Liu, Yu Qian, Shao-Wu Zhang

**Affiliations:** ^1^ Institute of Physics and Optoelectronics Technology, Baoji University of Arts and Sciences, Baoji, China; ^2^ Key Laboratory of Information Fusion Technology of Ministry of Education, School of Automation, Northwestern Polytechnical University, Xi’an, China

**Keywords:** sequence alignment, sequence mapping, single molecular sequencing, third-generation sequencing, long noisy reads

## Abstract

With the rapid development of single molecular sequencing (SMS) technologies such as PacBio single-molecule real-time and Oxford Nanopore sequencing, the output read length is continuously increasing, which has dramatical potentials on cutting-edge genomic applications. Mapping these reads to a reference genome is often the most fundamental and computing-intensive step for downstream analysis. However, these long reads contain higher sequencing errors and could more frequently span the breakpoints of structural variants (SVs) than those of shorter reads, leading to many unaligned reads or reads that are partially aligned for most state-of-the-art mappers. As a result, these methods usually focus on producing local mapping results for the query read rather than obtaining the whole end-to-end alignment. We introduce kngMap, a novel *k*-mer neighborhood graph-based mapper that is specifically designed to align long noisy SMS reads to a reference sequence. By benchmarking exhaustive experiments on both simulated and real-life SMS datasets to assess the performance of kngMap with ten other popular SMS mapping tools (e.g., BLASR, BWA-MEM, and minimap2), we demonstrated that kngMap has higher sensitivity that can align more reads and bases to the reference genome; meanwhile, kngMap can produce consecutive alignments for the whole read and span different categories of SVs in the reads. kngMap is implemented in C++ and supports multi-threading; the source code of kngMap can be downloaded for free at: https://github.com/zhang134/kngMap for academic usage.

## 1 Introduction

Single molecular sequencing (SMS) developed by Pacific Biosciences and Oxford Nanopore Technologies has been increasingly applied in DNA sequencing studies since its emergence (Ono et al., 2012; Rhoads and Au, 2015). Compared to next-generation sequencing (NGS), SMS can produce considerably longer reads with less sequencing bias and lower cost (Yang et al., 2017; Marchet et al., 2019). Recently, the N50 and maximum lengths of the reads generated by the MinION nanopore sequencer can achieve more than 100 kbp and 1 Mbp, respectively (Chen et al., 2021). Such long read lengths offer new solutions to bioinformatic questions that are hard to be resolved by NGS, such as the *de novo* genome assembly, genome resequencing, structural variant (SV) discovery, and transcriptome analysis (Stöcker et al., 2016; Wei and Zhang, 2018; Liu et al., 2019; Cao et al., 2021).

Read mapping is to find the possible genomic origins of reads by aligning them against a reference genome. It has become one of the most basic and computing-intensive step in downstream pipelines for SMS dataset analysis (Zhang et al., 2018). However, since the SMS reads have a higher error rate (∼15%) than the NGS reads, most of the read mapping methods developed for NGS short read data, such as Bowtie (Langmead et al., 2009), SOAP3 (Liu et al., 2012), CloudBurst (Michael, 2009), and FEM (Zhang et al., 2018), are not suitable to handle with SMS reads. Thus, a growing number of long noisy read mapping approaches specially designed for SMS long reads have been proposed during the past decade.

Broadly speaking, most existing mapping methods or tools for SMS reads adopt the typical seed-chain-align strategy (Liu et al., 2016a). In brief, they first find the matched *k*-mers (also called seeds) of query reads to the reference genome; then, the candidate regions (also called alignment skeleton) for aligning in the query read and the reference genome are chosen based on the seeds. Finally, the local subsequences surrounding the seeds are base-to-base aligned to compose the final read alignment with the seeds. Based on the index technique of seed searching, SMS mappers can be categorized into three groups: Burrows–Wheeler Transform full-text minute-space (BWT-FM) index-based (Langmead and Salzberg, 2012), hash table index-based, and short read aligner-based methods.

BWT-FM index-based methods find the matched seeds by constructing the BWT-FM index of the reference genome; such methods include BLASR (Chaisson and Tesler, 2012), BWA-MEM (Li, 2013), lordFAST (Haghshenas et al., 2018), and smsMap (Wei et al., 2020). BLASR (Chaisson and Tesler, 2012) initially builds the BWT-FM index of the genome to find short exact matches; then, the candidate aligned region is generated by using sparse dynamic programming (SDP), and the final detailed alignment within the area defined by SDP is performed by dynamic programming. BWA-MEM (Li, 2013) first searches the supermaximal exact matches according to the BWT-FM index of the reference and detects a group of seeds that are colinear and close to each other by a chaining algorithm, which then extends the seed with a banded affine-gap-penalty dynamic programming. lordFAST (Haghshenas et al., 2018) selects the candidate alignment regions in the genome using the longest matches identified by the BWT-FM index; then, the base-to-base alignment between consecutive seeds is obtained by performing dynamic programming. Recently, the smsMap (Wei et al., 2020) mapper proposed by us also utilizes the BWT-FM index to quickly find the matches in the reference genome and then locates the best starting positions in genome and query read by defining a credibility function. Finally, the detailed alignment result is obtained with the column reduction banded alignment method.

Hash-based mapping methods search the matched seeds through a hash table index of the genome. YAHA (Faust and Hall, 2012) utilizes a hash table index to store the *k*-mer locations in the reference and then extends seeds to generate fragments that contain contiguous matching bases between the query sequence and the reference genome. Next, it combines the fragments to select potential regions for alignment; lastly, it completes the full alignment by applying a modified version of Smith–Waterman algorithm to the unmatched regions. rHAT (Liu et al., 2015) starts to build the regional hash table (RHT) of the genome; then, the matches of the *k*-mers within the query read are retrieved through RHT and a direct acyclic graph is built to compose the skeleton of the alignment. At the end, unaligned pairs of segments in the skeleton are aligned with the banded Smith–Waterman algorithm. GraphMap (Ivan et al., 2016) implements the *q*-gram seeding strategy that allows for fast lookup of inexact matches by constructing a hash index of the reference sequence, which then generates alignment anchors through a fast graph-based ordering of seeds and extends anchors to achieve final alignments. minimap2 (Li, 2018) indexes minimizers in a hash table that allows for fast lookup of exact matches and then identifies colinear anchor sets; final base-level alignments are obtained by applying dynamic programming to regions between adjacent anchors. conLSH (Chakraborty and Bandyopadhyay, 2020) computes context-based locality-sensitive hashing values of genomes to facilitate seed search and then generates a series of sites for candidate alignment after extension; finally, it produces the best possible alignment results by applying the sparse dynamic programming-based approach. Later, S-conLSH (Chakraborty et al., 2021), an improved version of conLSH, was developed by introducing the spaced context-based locality-sensitive hashing for mapping long noisy SMS reads.

Short read aligner-based methods retrieve matches by using extant mappers of short reads. For example, LAMSA (Liu et al., 2016b) first looks for long approximate matches through short read aligner GEM (Marco-Sola et al., 2012), finds a set of possible alignment skeletons, and fills the gaps within the skeletons to generate valid alignments for the whole read. NGMLR (Sedlazeck et al., 2018) identifies similar segments in read and genome by short aligner of NextGenMap (Sedlazeck et al., 2013); then, it extracts the sub-sequences in read and reference to compute a pairwise sequence alignment using a convex gap cost model. Lastly, it reports the set of linear alignments with the highest joint score as the mapping results.

With the rapid development of SMS sequencing technologies, read length is continuously increasing; these longer reads could more frequently span the breakpoints of SVs than those of shorter reads (Kolmogorov et al., 2019; Ren et al., 2021). This may greatly influence read alignment since most state-of-the-art mappers do not consider the SV events, or few methods were designed for handling relatively small variants. Meanwhile, when the matched seeds are diversely distributed in some read parts caused by high sequencing errors, most extant aligners are incapable of obtaining the alignment of the read, leading to many unaligned reads or reads that are partially aligned. As a result, these methods usually focus on producing local mapping results for the query read rather than obtaining the whole end-to-end alignment, leading to a low mapping sensitivity.

To address the aforementioned issues, in this study, we propose a *k*-mer neighborhood graph mapper (named kngMap), a novel long-read mapping algorithm which is specifically designed to improve mapping sensitivity and deal with SV events. Overall, kngMap works in four main stages. It initially constructs a searching index for the reference genome to quickly find matched *k*-mers for query reads. Such matches are then used to construct a *k*-mer *d*-neighborhood graph where matched *k*-mers are viewed as vertices and each pair of matched *k*-mers is connected by a direct edge. Third, a high quality of alignment skeleton is identified by designing a chaining approach. At the end, each unaligned gap in the alignment skeleton is classified into several categories of SV events and filled with a specific alignment method according to its category. The whole read alignment is accomplished by integrating the skeletons and the alignments of the gaps. We benchmarked kngMap on simulated dataset reads with different types of SVs and real-life datasets generated from PacBio SMRT and Oxford Nanopore platforms. The experimental results demonstrated that kngMap has superior ability in terms of base-level sensitivity and end-to-end alignment, which can produce consecutive alignments for the whole read; meanwhile, it can deal with different categories of SVs in the reads.

## 2 Methods

**FIGURE 1:**
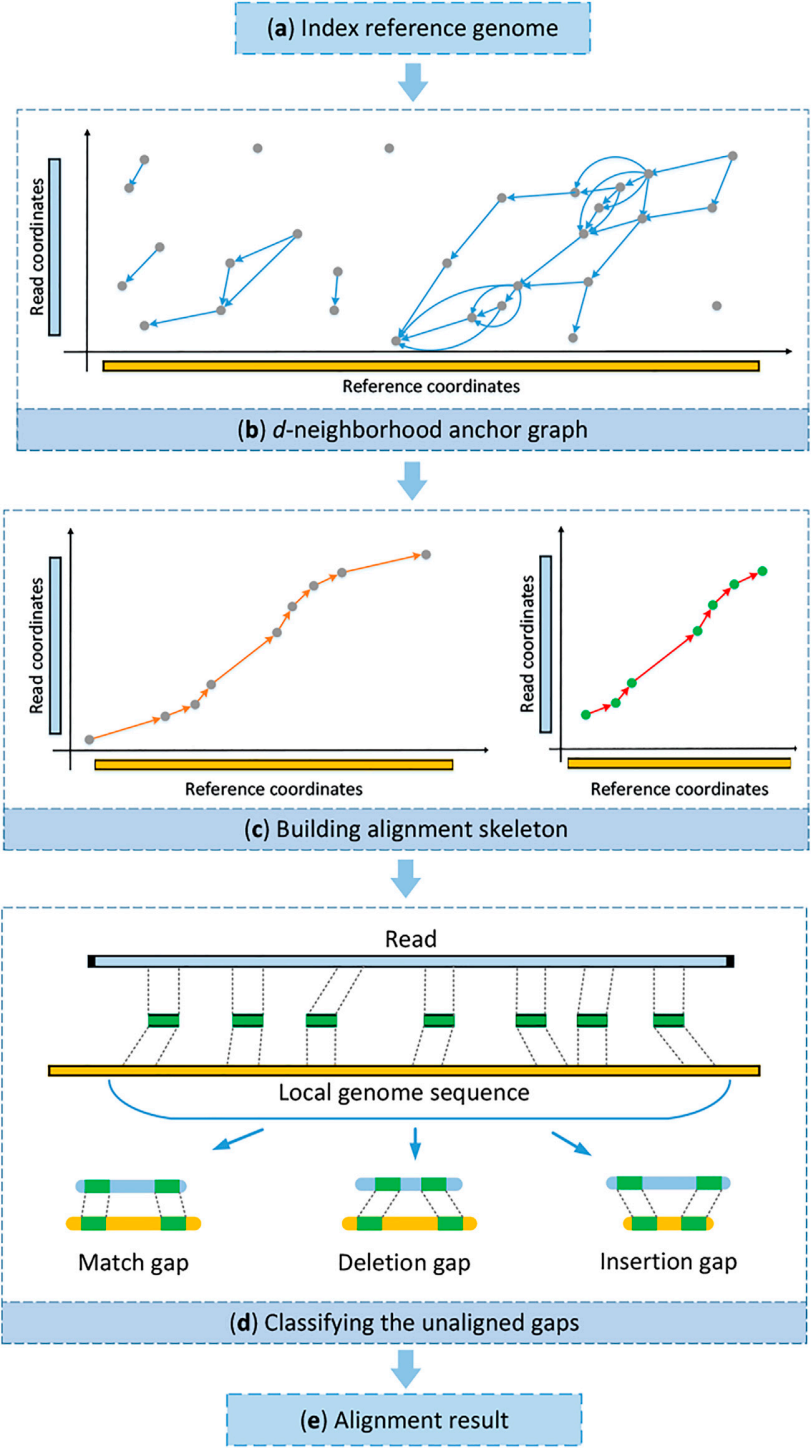
Schematic representation of stages in kngMap. **(A)** Building a searching index of the reference genome. **(B)** Constructing a *k*-mer *d*-neighborhood graph where matched *k*-mers (also called anchors) are viewed as vertices and each pair of matched *k*-mers is connected by a direct edge based on the positions in genome and the query read. **(C)** Building and refining the high quality of alignment skeletons by designing a chaining approach. **(D)** Classifying the unaligned gaps between pairs of consecutive anchor matches in the selected chain into several categories of SV events and handling each of them with a specific alignment method according to its category. **(E)** Generating the detailed base-to-base alignment by performing dynamic programming between consecutive anchor matches in the selected chain.

**FIGURE 2:**
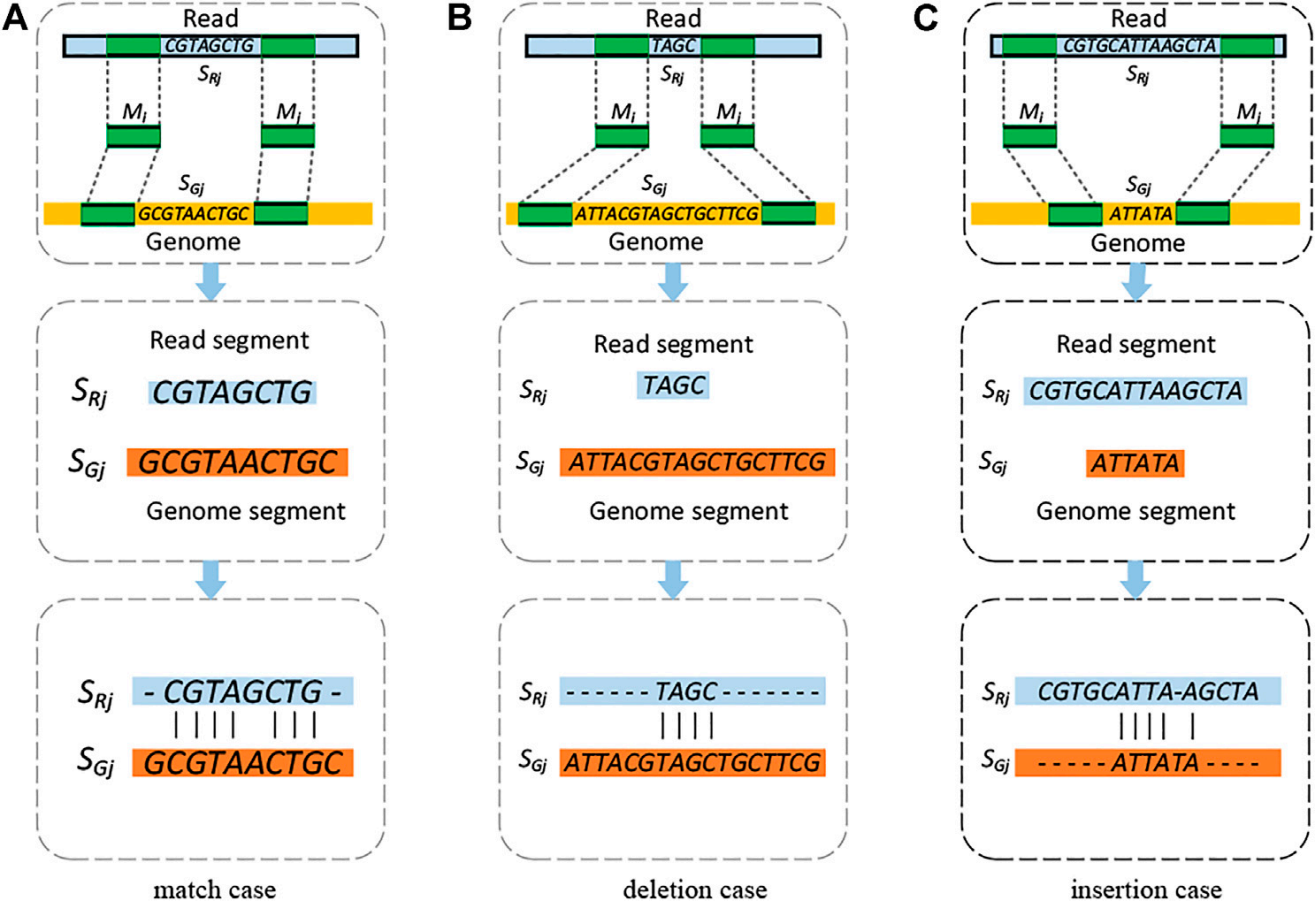
Schematic illustration of the three categories of unaligned segments in the read and genome. **(A)** Match, **(B)** deletion, and **(C)** insertion. In the figure, the light blue, yellow, and green bars present the read, the reference genome, and the anchored *k*-mers, respectively. The dashed lines connecting the anchored *k*-mers indicate the matched positions of the *k*-mers on the reference genome and the read. The classification conditions are based on the lengths of pair of unaligned segments. The second and third rows give an example on how to generate the alignment for each case.

**FIGURE 3:**
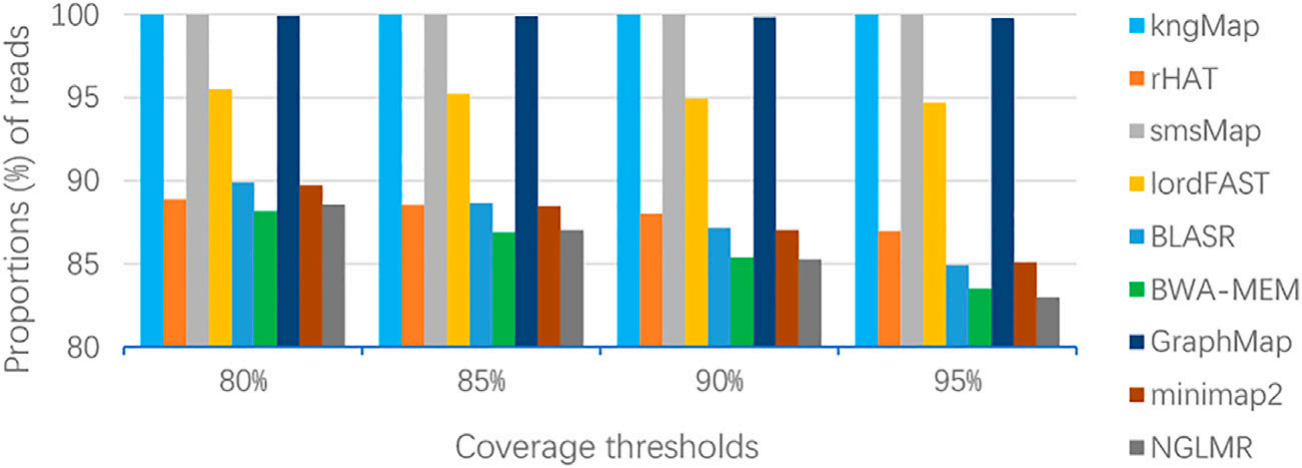
Consecutiveness alignment of different methods on the *A. thaliana* dataset. The percentage values labeled on the horizontal axis are the thresholds of the proportion of covered bases, that is, *c*_*i*_ (*i* = 1,2,3,4), for considering if the alignment result for a read is consecutive. The vertical axis bars indicate the proportions of reads consecutively mapped by different mappers. kngMap always achieved 100% consecutive alignments with all thresholds, while GraphMap achieved 99.91%, 99.88%, 99.84%, and 99.77% consecutive alignment at 80%, 85%, 90%, and 95% coverage thresholds, respectively. Therefore, kngMap is superior to GraphMap in providing better consecutive alignments. Each bar also corresponds to a value line in Supplementary Table S10.

The main motivation of kngMap is to efficiently improve mapping sensitivity and simultaneously have a superior ability of dealing with SVs for long reads generated by SMS sequencing technologies. The underlying design principle is to effectively find one high-quality alignment skeleton in the reference genome for each read, even though the read contains SVs and more sequencing errors, before the costly procedure of base-to-base alignment to the reference genome. An overview of the kngMap algorithm is depicted in Figure 1. kngMap mainly includes four stages: 1) building a searching index of the reference genome in advance, which is used to quickly find matched *k*-mers for a query read Figure 1A; 2) constructing a *k*-mer *d*-neighborhood graph where matched *k*-mers are viewed as vertices and each pair of matched *k*-mers is connected by a direct edge based on the positions in genome and the query read Figure 1B; 3) building and refining the high quality of the alignment skeleton by designing a chaining approach Figure 1C; and 4) classifying the unaligned gaps between pairs of consecutive matched *k*-mers in the alignment skeleton into several categories of SV events and handling each of them with a specific alignment method according to its category Figure 1D. A more detailed illustration of each step is provided later.

### 2.1 Constructing Reference Genome Index

In order to quickly find the occurrence positions of a *k*-mer (subsequence with length of *k*) in the reference genome, a lookup index for the reference genome is usually needed to be built. Currently, two superior index techniques, BWT-FM index (Lippert, 2005) and hashing table (Ning et al., 2001), are successfully used by most extant mapping methods (Lindner and Friedel, 2012). BWT-FM index allows long reference genome to be searched efficiently with low memory usage (Hayashi and Taura, 2013). Hash table takes linear time to find the positions in the genome when a certain *k*-mer is given (Berlin et al., 2015). Compared with BWT-FM index, the indexing speed based on the hash table is faster (Li, 2016; Prezza et al., 2016), but it will consume more computational memory. Both index techniques have their own advantages (Alser et al., 2021). Therefore, kngMap utilizes these two strategies [the BWT-FM technique implemented in combined-index (Haghshenas et al., 2018) and the hash index implemented in minimizers (Li, 2016)] to construct the index for the reference genome (the default index module is hash).

### 2.2 Generating *k*-Mer *d*-Neighborhood Graph

Once the index of the reference genome is constructed, all the matches of the *k*-mers and their matched positions for a query read can be retrieved through the genome index; these matches are subsequently used to form a *k*-mer *d*-neighborhood graph. Specifically, given a set of long reads, kngMap constructs the *d*-neighborhood anchor graph for each read at a time in two steps as follows:

#### Step 1: Extracting the Matched k-Mers

KngMap extracts all *k*-mers for one query read and finds the matched positions through the reference genome index built in the aforementioned stage. Each of the matches [also called anchors in some references (Chaisson and Tesler, 2012; Haghshenas et al., 2018)] of the *k*-mers can be denoted as a tuple: M(x,y,l) , where *x* and *y* are the matched positions on the read and the reference genome, respectively, and *l* is the length of the match (here *l* = *k*). In other words, M(x,y,l) indicates that the substring interval [*x* − *k* + 1, *x*] on the reference matches the interval [*y* − *k* + 1, *y*] on the query exactly.

#### Step 2: Generating the Anchor d-Neighborhood Graph

A *d*-neighborhood anchor graph is formed according to the list of anchors ordered by their positions on the reference genome and then on the read. In this graph, each anchor is viewed as nodes; two nodes (*V*_*i*_ → *V*_*j*_) are connected with a direct edge if the pair of vertices meets the following condition:

$$\{\begin{array}{l} V_{i}\left(x\right)-V_{j}\left(x\right)\leq d,\;x\;is\;the\;node\;position\;in\;the\;genome\\ V_{i}\left(y\right)-V_{j}\left(y\right)\leq d,\;y\;is\;the\;node\;position\;in\;the\;query\;read \end{array},$$ (1)

where $V_{i}\left(x\right)$ is the position of node *i* in the reference genome, $V_{j}\left(x\right)$ is the position of node *j* in the query read, and *d* is a constraint parameter which is applied to model the maximum distance between two anchors in the alignment for a read. The edge direct (from *V*_*i*_ to *V*_*j*_) denotes that *V*_*i*_ is the ancestor (also called precursor or predecessor) node of *V*_*j*_. The setting of parameter *d* is motivated by the fact that, given a certain error rate, the distance between two anchors on the read can be modeled by a geometric distribution (Chaisson and Tesler, 2012). In kngMap, this property is utilized to describe the maximum allowed distance on the read between two vertices connected by an edge in considering the error rate of the read. Theoretically, two continuous *k*-mers in an error-free read are also matched in the continuous positions in the genome (Supplementary Figure S1). However, in fact, with the sequence errors derived from SMS sequencing, they can be matched with two discontinuous positions in the genome (Supplementary Figure S2), and the distance between these two discontinuous positions is usually lower than *d*. Under this circumstance, *d* can prevent a lot of unnecessary connections; furthermore, it can facilitate building the skeleton in the next step due to the fact that some vertices are lack of precursors. Here, we set d=λ×L(r) , which is a variable value that depends on the length of the query read, where λ (default value is 1.2) is an empirical value according to the length ratio (defined as the length of the aligned region in reference divided by the length of the aligned region in read) distribution of the aligned results shown in Supplementary Figure S3. With the setting of *d*, it has a high probability that the distance between two neighboring true positive nodes can be successfully connected with an edge.

### 2.3 Generating the Skeleton of Alignment

Building the skeleton of alignment is a core step for a mapping algorithm since it will dramatically facilitate the base-to-base alignment. The alignment skeleton (Liu et al., 2021) is a set of nonoverlapping anchors that have the highest possibility to form the candidate aligned region in the read and reference genome. In consideration of the potential breakpoints within reads, we proposed a specific chaining strategy to find the skeleton of alignment in the following two steps:

#### Step 1: Generate the Initial Alignment Skeleton

KngMap finds the optimal path connecting from *V*_*start*_ to *V*_*end*_, which maximizes the total number of matched bases as the skeleton of alignment. This is implemented by scoring the vertices in the *d*-neighborhood graph with the following recurrence equation:

$$score\left(V_{i}\right)=\{\begin{array}{l} \underset{V_{j}\in pre\left(V_{i}\right)}{\max}\left\{score\left(V_{j}\right)+\alpha \right\},\;\;if\;pre\left(V_{i}\right)\neq \emptyset \\ 0,otherwise \end{array},$$ (2)

where $score\left(V_{i}\right)$ is the score assigned to the vertice *V*_*i*_ in the graph, $pre\left(V_{i}\right)$ is the precursor set of *V*_*i*_, and α (default value is 1) is the reward score between the two vertices linked with an edge. With Eq. 2, each vertex can find a precursor maximizing its score, and the node (*V*_*end*_) with the highest score among all nodes is regarded as the ending node. As a result, the path from *V*_*end*_ to a starting node (*V*_*start*_ backtracking from *V*_*end*_ to a node without precursor) formulates the initial alignment skeleton.

From Eq. 2, we can see that the parameter of reward score ( α ) is set with a constant value; this is critical to deal with some regions containing SV events (e.g., longer insertion and deletion gaps) or high sequencing errors, especially when the lengths of these regions are long. By setting to a constant value, two anchors can successfully span the region with SV or high sequencing errors, as shown in Supplementary Figure S4; otherwise, two chains will be generated if a low score is assigned to an anchor when the distance between this anchor and its ancestor is too large (Li, 2018). Therefore, with a constant scoring parameter α , it can make sure that the edge connecting the two matches flanking the SV or regions with high errors can be detected. Thus, the scoring scheme in Eq. 2 helps recover longer insertions and deletions.

#### Step 2: Refining the Alignment Skeleton

Next, kngMap refines the skeleton by pruning the initial alignment skeleton. This pruning process aims to avoid some false positions caused by sequencing errors or repeat regions. As shown in Figure 1C, the skeleton is generated by the previous step. Obviously, the two ending anchors in Figure 1C should not be considered for downstream detail alignment. Thus, an increased refining procedure is carefully designed to deal with such situations. In the skeleton, kngMap only chooses the chains (a subset of the anchors in the initial skeleton) with the highest increased score using the following equation:

$$\arg\;\underset{V_{i},\;V_{j}}{\max}\left\{score\left(V_{i}\right)-score\left(V_{j}\right)\right\},\;V_{i}\left(x\right)-V_{j}\left(x\right)<l,$$ (3)

where *l* is the constraint length parameter (default value *l* = *len*(*r*)), which guarantees that the increased score is calculated in a fixed window length in the path. This setting is motivated by observing that the anchors of a read alignment are fallen in the region with a length of *l*, which is found by the statistics based on the alignment results (Supplementary Figure S5). After the pruning, the two nodes that have the maximum increased scores are selected as the final starting and ending nodes (denoted as ${V^{\prime }}_{start}$ and ${V^{\prime }}_{end}$ ), and the path between ${V^{\prime }}_{start}$ and ${V^{\prime }}_{end}$ is regarded as the final refined skeleton of alignment.

### 2.4 Filling the Gaps Between Anchors

With the refined skeleton of alignment, the numbers of pairs of unaligned segments can be directly partitioned by the anchors, which is shown in Supplementary Figure S6. In order to obtain the whole base-to-base alignment of the read, kngMap distinguishingly performs a detailed alignment between each pair of unaligned segments. As illustrated in Supplementary Figure S6, the skeleton (from *M*_*1*_ to *M*_*7*_) partitions the read and the reference into eight paired segments. Each pair of unaligned segments, that is, (*S*_*Ri*_, *S*_*Gi*_), *i* = 1, 2, 3, 4, 5, 6, 7, will be categorized into one of the following conditions, which carefully take the SV into consideration according to the length of segments.

1) Match case: $\left|L\left(S_{Ri}\right)-L\left(S_{Gi}\right)\right|\leq L\left(S_{Ri}\right)\times \mu$ .

2) Deletion case: $L\left(S_{Ri}\right)-L\left(S_{Gi}\right)<L\left(S_{Ri}\right)\times \mu$ .

3) Insertion case: $L\left(S_{Ri}\right)-L\left(S_{Gi}\right)>L\left(S_{Ri}\right)\times \mu$ .

Here, *S*_*Ri*_ and *S*_*Gi*_ are the paired partitioned subsequence in the read and genome, respectively, $L\left(S_{Ri}\right)$ is the length of *S*_*Ri*_, $L\left(S_{Gi}\right)$ is the length of *S*_*Gi*_, and μ is a user-defined parameter categorizing the cases. The parameter of μ is used to model specific categories of SVs and the default value of μ is set with 2, which is an empirical value based on the alignment statistics (Supplementary Figure S7). A schematic illustration of the three categories of unaligned segments is shown in Figure 2.

KngMap then fills the paired segments within the skeletons to solve the breakpoints of SVs and generate valid alignments for the whole read. Each of the gaps is filled by one of the following strategies according to its category. For the match case, as shown in Figure 2A, kngMap directly performs an end-to-end alignment based on the NW (Needleman–Wunsch) algorithm between the read segment (*S*_*Rj*_) and the reference genome segment (*S*_*Gj*_) to fill the gap; an example of dealing with this match case is also provided in Figure 2A. For a deletion case (Figure 2B), there is a potential deletion in *S*_*Rj*_; moreover, we do not know if the SV position exists. In order to effectively deal with such cases, a semi-global alignment is performed, which is beneficial for handling multiple breakpoints within *S*_*Gj*_. For the example in Figure 2B, there are two deletions within the *S*_*Rj*_; the semi-global alignment can effectively recognize them since it can find the most similar regions in *S*_*Gj*_ for *S*_*Rj*_. In the implementation of the semi-global alignment, the genome segment (*S*_*Gj*_) was chosen to be the target sequence and read segment (*S*_*Rj*_) to be the query sequence as the alignment gaps at the query start and end are not penalized. Similarly, for an insertion case (Figure 2C), there is a potential insertion in *S*_*Rj*_, and we do not know if its positions exist. For this case, the semi-global alignment is also applied to recognize the insertion. The read segment (*S*_*Rj*_) was chosen to be the target sequence and genome segment (*S*_*Gj*_) to be the query sequence in the implementation of the semi-global alignment.

### 2.5 Extending the Boundaries of the Skeletons

All the operations mentioned previously fill the inner gaps of the skeleton, which are anchored by two matched *k*-mers. For the outer boundaries of the refined skeleton, that is, the two pairs of unsigned segments at the starts and ends of the skeletons as shown in Supplementary Figure S8, kngMap assumes that these boundaries of the genomic region is SV-free and directly extends the boundaries by performing a modified global alignment to obtain the base-to-base alignment. To be more specific, for the alignment between the suffix of the read and the reference following the last anchor, kngMap first extracts the *S*_*Rend*_ as the query sequence and the *S*_*Gend*_ (with two times length than *S*_*Rend*_) as the target sequence. Then, the modified global alignment (similar to the global NW alignment method but with a small twist gap at the query end that is not penalized) is performed for *S*_*Rend*_ and *S*_*Gend*_; the modified global alignment can find out how well *S*_*Rend*_ fits at the beginning of *S*_*Gend*_. A toy example of it is illustrated in Supplementary Figure S8. Similarly, the alignment between the prefix of the read and the reference prior to the first anchor can be computed in an identical fashion. Finally, the whole read alignment is accomplished by integrating the skeletons and the alignments of the gaps Figure 1E. Overall, the pseudo-code for the kngMap method procedure is described in **Algorithm 1**:

Algorithm 1 kngMap Algorithm.

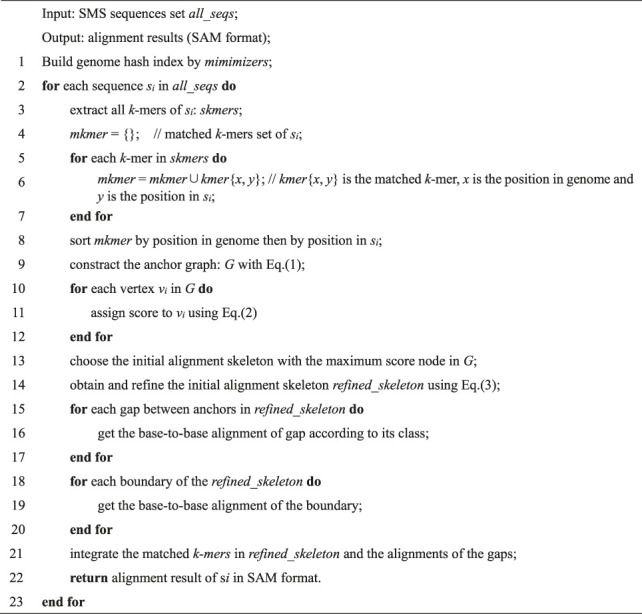

## 3 Results

The performance of kngMap was evaluated against 10 state-of-the-art long read aligners: BLASR (v) (Chaisson and Tesler, 2012), minimap2 (v2.12-r829) (Li, 2018), BWA-MEM (v0.7.17-r1194) (Li, 2013), GraphMap (v0.5.2) (Ivan et al., 2016), rHAT (v0.1.2) (Liu et al., 2015), NGMLR (v0.2.7) (Sedlazeck et al., 2018), lordFAST (v0.0.9) (Haghshenas et al., 2018), smsMap (Wei et al., 2020), conLSH (v0.0.1) (Chakraborty and Bandyopadhyay, 2020), and S-conLSH (v0.0.1) (Chakraborty et al., 2021). All methods were benchmarked on simulated and real-life SMS sequencing datasets. All the experiments were performed on a Linux machine server running Ubuntu 14.04 system equipped with two twelve-core (two threads per core) Intel Xeon Gold 5118 CPU @ 2.30GHz and 256 gigabytes (GB) RAM. The run command lines and parameters of each mapping tools are available in Supplementary Table S1.

It is to be noted that with the rapid development of SMS sequencing technology, more than 99% of the raw sequencing data produced by the SMS sequencing platform has a read length of 1,000 bp or longer (Haghshenas et al., 2018). Herein, reads with lengths shorter than 1,000 bp were filtered so that the remaining reads that have 1,000 bp or longer are aligned in the following experiments.

### 3.1 Experiment on Simulated Datasets

#### 3.1.1 Simulation Without Structural Variations

We first adopted the simulated sequence datasets without structural variations (SVs) to evaluate the performance of kngMap algorithm against the aforementioned mapping methods. The simulated datasets were generated by PBSIM2 (Ono et al., 2020), a new simulator that can capture the characteristics of errors in reads for both PacBio and Nanopore sequencer. The *H. sapiens* (CHM1) genome sequences were downloaded from NCBI (assembly accession: GCA_000306695.2) and fed into PBSIM2 software using default error parameters (8% deletions, 7% insertions, and 1% substitutions). As a result, 100,000 simulated sequences with an average read length of 10,157 bp were generated by PBSIM2. The detailed running commands of PBSIM2 and some statistics of simulated datasets can be found in Supplementary Tables S2 and S3.

For the simulated dataset, we know exactly the true mapped region and bases in the reference genome for each read, so the correctly mapped reads (CMRs) and correctly mapped bases (CMBs) were applied to investigate the overall quality of the alignments. A read *r* is called CMR if this read is aligned to the derived genome with the correct strand, and the overlap between the aligned subsequence on the reference and the true mapping subsequence has at least *p* bases (*p* = 0.9 × *len*(*r*), where *len*(*r*) is the read length of *r*). A base is called CMB if it is located within *T* bp (here *T* = 5) of the corresponding truth position on the genome. We further compared the aligned coverage (proportion of aligned bases for one read) to assess the alignment for different methods. A method with higher aligned coverage means that it can align more bases to the reference genome for a read. In addition, base-level sensitivity and precision (Marco-Sola et al., 2012) are used to evaluate the performance of different mappers for the simulated sequence dataset. Sensitivity is defined as the number of correct matched bases divided by the total number of bases, and precision is defined as the number of correct matched bases divided by the number of mapped bases.

Based on the aforementioned metric definition, Table 1 shows the evaluation result of kngMap, rHAT, smsMap, lordFAST, BLASR, BWA-MEM, GraphMap, minimap2, NGMLR, conLSH, and S-conLSH on the simulated datasets without structural variations. As can be seen from Table 1, kngMap not only correctly mapped more reads than any other mapper but also correctly aligned 99.82% of the total number of bases, which improves the sensitivity by 0.1%–2.4% over its competitors, except the conLSH and S-conLSH methods. For the base sensitivity and precision in Table 1, we can observe that kngMap still performed the best, indicating that most bases are correctly mapped in the alignments generated by kngMap. It is worth noting that the alignment results of conLSH and S-conLSH are obviously worse than those of other methods, so we do not show the results of these two methods in the following comparisons.

**TABLE 1:** Mapping results of different mapping tools on the simulated human dataset. This dataset contains 10,000 reads and 1.015 billion bases. The best results are labeled with a bold typeface.

Method	CMR	CMB	Aligned coverage (%)	Sensitivity (%)	Precision (%)
kngMap	**99,711**	**1,013,921,165**	**100**	**99.82**	99.82
rHAT	99,516	1,008,128,007	99.95	99.25	99.59
smsMap	98,747	993,435,787	99.98	97.80	97.80
lordFAST	99,709	1,012,991,874	99.99	99.73	99.85
BLASR	99,648	1,010,806,725	99.72	99.51	99.77
BWA-MEM	99,603	1,010,085,677	99.88	99.44	99.63
GraphMap	98,594	1,001,398,553	99.99	98.85	98.72
minimap2	99,698	1,013,427,775	99.89	99.77	99.90
NGMLR	97,207	990,054,543	99.83	97.47	98.67
conLSH	105	1,207,640	99.74	0.11	0.12
S-conLSH	2,349	115,168	99.98	0.01	0.01

Importantly, kngMap achieved 100% aligned coverage, demonstrating that all mapped reads by kngMap are completely aligned, while the alignment by other aligners cannot cover the whole sequence, which means that these methods always output local mapping results and cannot obtain the end-to-end alignment for the whole read. After checking the details of the alignments, soft clipping at the starting or ending of sequences is usually produced by other methods. These results in aligned coverage explain that kngMap has a higher base-level sensitivity. This 100 percent coverage achieved by kngMap is beneficial for SMRT data analysis since higher aligned coverage is a key requirement for mapping tools and downstream mapping-based applications (Schmieder and Edwards, 2012; Laver et al., 2015).

In addition, we inspected the reads of the simulated human dataset which were incorrectly aligned by kngMap and found that 255 (88.29%) of the reads have a higher similarity than the original similarity simulated by the PBSIM2. To give an example, Supplementary Material (named as *S9_46798.txt*) reports the alignment result for one simulated read with a length of 6,692 bp; the mapped alignment identity obtained by kngMap is 90.74%, while the simulated identity generated by PBSIM2 is 87.50%, which is lower than the similarity produced by kngMap. Therefore, this further indicates that kngMap can find the most similar mapped region in the reference sequence for each query read.

#### 3.1.2 Simulation in the Presence of Structural Variations

For assessing the ability of handling SVs for different methods, a simulated sequence dataset with different types of SVs was generated. Specifically, 16 SVs (i.e., five insertions, eight deletions, and three inversions) with different sizes were first added into the reference chr1 (chromosome 1 of CHM1) genome by the RSVSim (Bartenhagen and Dugas, 2013) simulator, an R package tool for the simulation of SVs with various sizes and SV types in any genome available as the FASTA file. Then, the chr1 genome of CHM1 with SVs was fed into PBSIM2 to produce the final simulation read set. Among the simulated reads, a total of 129 reads cover the SV breakpoints. The detailed SVs and its breakpoints are listed in Supplementary Table S4.

Here, we provide the number of mapped reads that span SVs (#SVs) to evaluate the performance of different mapping tools. For one mapped read, if the start and end alignment coordinates in the genome cover the actual simulated breakpoints, we consider this read spanning SVs. Specifically, each of the breakpoints described by the ground truth in the genome can be denoted as a tuple: $BP_{i}^{T}\left(PG_{start}^{T},\;PG_{end}^{T}\right),\;i=1,\;2,\;\cdots \;,\;N_{BP}^{T}$ , where $PG_{start}^{T}$ and $PG_{start}^{T}$ are the starting and ending positions of the breakpoint on the reference genome, respectively, and $N_{BP}^{T}$ is the total number of ground truth breakpoints. Similarly, each of the alignment coordinates mapped by a mapping tool for a simulated read with SV can be denoted as a tuple: $MC_{i}\left(MG_{start},\;MG_{end}\right),\;i=1,\;2,\;\cdots \;,\;N_{read}^{SV}$ , where $MG_{start}$ and $MG_{end}$ are the starting and ending mapped coordinates on the reference genome, respectively, and $N_{read}^{SV}$ is the total number of reads covering the SV breakpoints ( $N_{read}^{SV}$ = 129).

With the ground truth breakpoints and the mapped coordinates obtained by a mapping tool, we can assess the number of ground truth breakpoints being recovered. For a certain read, a ground truth breakpoint, $BP_{i}^{T}\left(PG_{start}^{T},\;PG_{end}^{T}\right)$ , is considered being recovered, only if there is at least one mapped coordinate, $MC_{i}\left(MG_{start},\;MG_{end}\right)$ , meeting the following condition:

$$\{\begin{array}{c} MG_{start}<PG_{start}^{T}\\ MG_{end}>PG_{end}^{T} \end{array}.$$ (4)

Finally, the number of ground truth breakpoints being recovered from our kngMap and other seven mapping programs is listed in Table 2, from which we can see that kngMap can map more reads with SVs on the genome than the other approaches, which demonstrate that our kngMap is capable to handle the SV-spanning reads.

**TABLE 2:** Number of mapped reads that span SV breakpoints for different mapping methods.

	kngMap	smsMap	lordFAST	BLASR	BWA-MEM	GraphMap	minimap2	NGMLR
#SVs	122	97	119	114	87	119	115	103

Note: rHAT tool always appears as segmentation fault (core dumped) information for the simulated reads with SVs, so we did not show the results of rHAT.

### 3.2 Experiment on Three Real Datasets

In this experiment, three datasets (generating from PacBio SMRT and Oxford Nanopore platforms) of *A. thaliana*, *E. coli UTI89*, and *H. sapiens* (CHM1) were used to benchmark kngMap against other methods on real sequencing data. Among these datasets, *A. thaliana* and *H. sapiens* (CHM1) were generated from the PacBio SMRT platform, and *E. coli UTI89* was generated from the Oxford Nanopore MinION sequencer. The availability of these datasets and their reference genomes are provided in Supplementary Tables S5 and S6, respectively, and the detail statistics (e.g., read number and length distributions) related to these datasets are given in Supplementary Figure S7. In the absence of true mapping locations for these real-life datasets, the mappers were compared based on different metrics as follows.

First, the number of mapped reads, the number of mapped bases, the number of matched bases, and the alignment score are used to evaluate the performance of different mapping methods. The number of mapped reads (read-level) and bases (base-level) are two important metrics to evaluate mapping sensitivity for long read alignment since it could still be lack of information for downstream analysis if reads are unmapped or only partially aligned (Peng et al., 2015). The number of mapped bases is usually viewed as a metric in base-level sensitivity. The number of matched bases and the alignment score can reflect the quality of the reported alignments for each method (Haghshenas et al., 2018). Specifically, for each mapping of a read, the matched base is defined as the base which is mapped to the identical one in the reference genome, the alignment score is calculated with scoring parameters: match = +1, mismatch (including insertion, deletion, substitution, and unmapped/clipped cases) = −1, gap opening = −1, and gap extension = −1, and the sum of alignment scores of all mapped reads was calculated for each method. Table 3 reports the mapping results of nine methods for the *A. thaliana* dataset. Obviously, it can be seen that kngMap mapped the most reads and aligned the most bases, that is, kngMap mapped 21,182 reads and 185,924,663 bases, improving sensitivity by 3.5% and 4.3% over the closest competitor (lordFAST) in terms of mapped reads and mapped bases, respectively, and even more compared with other tools. For the matched bases and alignment score, kngMap still performed the best; more precisely, kngMap reports a 6.02 million higher number of matched bases and 4.32 million higher alignment score compared to the best runner-up (i.e., lordFAST). These results indicate that kngMap can not only provide more sensitive alignments in read-level and base-level sensitivity than other mappers but also achieved the best quality of the alignments. Similar results (Supplementary Tables S8 and S9) were replicated in two other real datasets (*E. coli UTI89* and *H. sapiens*) as well.

**TABLE 3:** Number of mapped reads, mapped bases, mapped matched bases, and the alignment score of nine methods on the real *A. thaliana* dataset. The best results are labeled with a bold typeface.

Method	Mapped reads	Mapped bases	Matched bases	Alignment score
kngMap	**21,182**	**185,924,663**	**170,116,753**	**144,880,696**
rHAT	21,139	161,930,990	149,098,181	104,998,955
smsMap	21,170	185,937,988	162,348,708	131,169,015
lordFAST	21,156	179,616,861	165,787,986	138,853,327
BLASR	20,951	170,669,368	156,421,685	122,279,677
BEA-MEM	20,987	168,716,088	157,879,323	125,312,363
GraphMap	20,088	175,859,786	161,472,579	139,464,799
minimap2	20,748	169,541,803	158,229,925	127,139,769
NGMLR	18,918	155,650,064	144,968,837	117,202,862

Next, we investigated the consecutive alignments, which can reflect the details of the alignments (Liu et al., 2015). To compare the consecutiveness of the alignments, we set four covering thresholds (i.e., $c_{1}=80\%$ , $c_{2}=85\%$ , $c_{3}=90\%$ , and $c_{4}=95\%$ ) to inspect the aligned proportion of the read which has at least one alignment covering at least *c*_*i*_ (*i* = 1,2,3,4) proportion of the whole read. If one alignment covers more than *c*_*i*_ of the read, the alignment is defined as the consecutive alignment at threshold *c*_*i*_. Figure 3 describes the results of the consecutive alignment for nine methods, and the detailed values shown in Figure 3 can be seen in Supplementary Table S10. From Figure 3, we can clearly see that kngMap and smsMap always achieved 100% consecutive alignments at all thresholds, indicating that kngMap and smsMap can achieve the end-to-end alignment for each read, which will facilitate downstream analysis in practice since each mapped read is completely aligned and not split aligned. GraphMap also achieved high consecutive alignment, while other methods, especially BWA-MEM and NGMLR, tend to produce more reads that have a large proportion of bases being clipped. Similar results can be found in Supplementary Figures S9 and S10 for the real dataset of *E. coli* and *H. sapiens*. The consecutive results in Figure 3 and Supplementary Figures S9 and S10 demonstrate that kngMap can provide better consecutive alignments, which is the main reason for the higher base-level sensitivity of kngMap.

**FIGURE 4:**
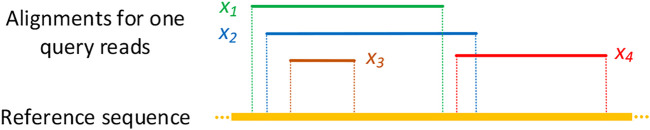
Examples of covering and non-covering alignment results. *x*_*1*_, *x*_*2*_, *x*_*3*_, and *x*_*4*_ are different alignment results reported by four mapping methods for the same query read. The dotted line indicates the mapped region in the reference genome sequence for the corresponding alignment result. In this figure, *x*_*1*_ and *x*_*2*_ alignments cover each other as they span the mapped regions on the reference genome that have at least 90% overlap. The alignments *x*_*1*_ and *x*_*2*_ cover *x*_*3*_ alignment but not the *x*_*4*_ alignment, while *x*_*3*_ does not cover *x*_*1*_ and *x*_*2*_ alignments. On the other hand, the alignment *x*_*4*_ does not cover either alignment *x*_*1*_, *x*_*2*_, or *x*_*3*_.

Furthermore, the agreement between different methods based on their alignment results was measured. Supposing that alignments *x*_*1*_ and *x*_*2*_ are two alignment results obtained by two mapping methods for a query read, we define that *x*_*1*_ has an agreement with *x*_*2*_ if and only if the mapped region on the reference genome covered by *x*_*1*_ overlaps with at least 90% of the mapped region on the reference genome covered by *x*_*2*_. Figure 4 describes some toy examples to illustrate the covering and non-covering alignments. As a result, the agreement alignments between each pair methods on the *A. thaliana* dataset are shown in Table 4. Each row in Table 4 denotes the percentage of the alignments produced by the corresponding method that covers alignments generated by other tools listed in the column. For example, among all mapped reads for kngMap and minimap2 in Table 4, kngMap covers 91.85% of the alignments produced by the minimap2 method, while minimap2 only covers 78.82% of the alignments generated by kngMap. Supplementary Tables S12 and S13 report the agreement between different methods on *E. coli* and *H. sapiens* datasets, respectively. We can see that the mapping results of kngMap have a high coverage of the alignments obtained by other mapping methods.

**TABLE 4:** Agreement of different alignment methods for the real *A. thaliana* dataset.

	kngMap	rHAT	smsMap	lordFAST	BLASR	BWA-MEM	GraphMap	minimap2	NGMLR
kngMap	-	79.98	76.72	84.59	72.16	77.03	87.59	78.82	74.21
rHAT	87.58	-	79.80	85.49	79.91	84.39	86.22	85.85	79.82
smsMap	89.07	79.30	-	85.67	78.23	77.44	86.38	79.12	74.36
lordFAST	88.32	81.45	78.29	-	75.25	80.00	86.63	81.14	75.58
BLASR	90.08	84.27	89.22	89.41	-	90.11	88.85	91.96	82.58
BWA-MEM	90.71	83.85	84.66	89.99	84.78	-	89.06	91.36	83.02
GraphMap	92.94	83.99	79.67	88.28	75.95	80.82	-	82.44	78.25
minimap2	91.85	84.93	85.06	90.03	86.47	91.61	89.68	-	83.75
NGLMR	95.62	89.91	89.42	93.46	90.55	94.44	94.59	96.34	-

Finally, we compared the performance of the tools on reads for which their alignments do not agree. The number of disagreeing alignments and average identity difference between the two methods for the inconsistent alignments are listed in Table 5. The numbers in each row indicate the number of reads for which the corresponding method reports alignments that do not cover alignments outputted by the other methods listed in the column. The positive/negative percentage in parentheses means that the average identity of the disagreeing alignments for the corresponding method is higher/lower than the average identity of the disagreeing alignments for the other methods listed in the column. For instance, there are 1,997 reads for which BLASR does not cover the alignments of kngMap. For those reads, BLASR produced alignments with an average of 5.83% lower identity than kngMap. On the contrary, there are 5,497 reads for which kngMap does not cover BLASR’s alignments. For those reads, on average, kngMap alignments have 24.74% higher identity than those of BLASR. This indicates that kngMap can find more similar alignments than BLASR. The performance of the tools on *E. coli* and *H*. *sapiens* reads is provided in Supplementary Tables S13 and S14. With a lack of the true mappings for the real dataset, the information in Table 5 and Supplementary Tables S13 and S14 are some extra support for the fact that the alignments of our kngMap are reliable.

**TABLE 5:** Performance of each pair methods on the *A. thaliana* dataset for which their alignments do not agree.

	kngMap	rHAT	smsMap	lordFAST	BLASR	BWA-MEM	GraphMap	minimap2	NGMLR
kngMap	-	4,014 (90.15)	4,852 (9.46)	3,054 (11.66)	5,497 (24.74)	4,496 (31.60)	1,414 (−1.22)	3,893 (31.91)	3,101 (40.99)
rHAT	2,524 (−30.04)	-	4,270 (−14.97)	2,917 (−19.06)	3,975 (−7.57)	3,070 (−11.22)	1809 (−27.80)	2,537 (15.07)	2034 (15.03)
smsMap	2,230 (−6.06)	4,258 (67.14)	-	2,920 (7.33)	4,299 (22.66)	4,503 (21.20)	1700 (−6.05)	3,907 (20.95)	3,089 (30.72)
lordFAST	2,383 (1.60)	3,812 (97.58)	4,591 (10.61)	-	4,996 (28.68)	4,016 (33.68)	1742 (−0.29)	3,553 (36.91)	2,921 (40.55)
BLASR	1997 (−5.83)	3,212 (50.74)	2,258 (−3.29)	2,184 (9.02)	-	2004 (3.31)	1,407 (−3.33)	1,508 (0.59)	1,609 (35.75)
BWA-MEM	1869 (1.55)	3,311 (56.90)	3,218 (2.43)	2053 (14.00)	3,092 (6.06)	-	1,330 (2.40)	1,570 (6.17)	1,489 (38.46)
GraphMap	1,388 (1.79)	3,164 (94.77)	4,083 (9.49)	2,336 (15.95)	4,765 (25.09)	3,785 (33.64)	-	3,341 (33.88)	2,918 (39.92)
minimap2	1,630 (2.58)	3,062 (57.86)	3,098 (2.53)	2041 (15.33)	2,765 (2.83)	1737 (5.80)	1,249 (−0.14)	-	1,490 (39.72)
NGLMR	819 (−21.91)	1899 (52.86)	2000 (−5.35)	1,229 (5.48)	1780 (−8.57)	1,407 (−10.16)	742 (−19.50)	641 (−24.43)	-

## 4 Discussion

For SMS read mapping, the seed-chain-align procedure (Lin and Hsu, 2020), as is used by most mapping methods, is a classical and effective strategy to obtain the superior alignment results since it can help to seek the alignment skeleton consisting of matched segment pairs between the long SMS read and the reference genome. Generally, the seed-chain-align procedure can be summarized as three phases: first, matched *k*-mers are found as seeds in one read and in the reference sequence. Then, a group of seeds that are colinear or close to each other is chained as the candidate alignment skeleton. Finally, the non-seed fragments within the alignment skeleton are being extended to generate the base-level alignment. In the stage of generating the alignment skeleton, these methods are usually difficult to capture the matches with long distance, especially for the read parts with SVs and high sequencing errors since that there are few or no matched *k*-mers in the parts. Thus, most methods failed to generate the alignment skeleton of the query read or just build an alignment skeleton that covers a small part of the read. As a result, these methods cannot obtain the alignment result or just report a local mapping result for the query read, other than obtaining the whole end-to-end alignment, leading to low mapping sensitivity and aligned coverage.

To address the aforementioned challenge, here, we developed kngMap to increase the mapping sensitivity and with 100% aligned coverage for long noisy alignment. kngMap is also a seed-chain-align method using the hashing index technique, compared with other methods. There are three main key features of kngMap: 1) kngMap proposes a scoring strategy in the chaining procedure to choose a group of anchors to form the initial alignment skeleton for each read, even in the situation that the read contains SVs or some regions that matched seeds are dispersedly distributed caused by high sequencing errors; 2) defining an increased credibility function to refine the alignment skeleton, and then a high quality of alignment skeleton is subsequently obtained; and 3) for each of the gaps within the skeleton, kngMap classifies it into one of three categories and implements a specific alignment strategy to fill the corresponding gaps. The scoring strategy and increased credibility function ensures that kngMap can effectively find the alignment skeleton for every query read, even in the situation that the matched seeds are dispersedly distributed in the reference genome. Thus, kngMap can obtain higher mapping sensitivity, that is, align more reads and bases. The last feature can guarantee that the whole end-to-end alignment is obtained, not local alignment achieved by other methods. Therefore, the aligned coverage of kngMap is higher than that of other methods. In addition, in order to further evaluate the performance of the kngMap for a higher error rate up to ∼15%, we applied the PBSIM2 simulator to generate another two simulated datasets with 20% (parameter: --accuracy-mean 20) and 25% (parameter: --accuracy-mean 25) error rates; the other parameter settings are similar to those given in Supplementary Table S2. Supplementary Table S14 shows the evaluation result of kngMap, rHAT, smsMap, lordFAST, BLASR, BWA-MEM, GraphMap, minimap2, and NGMLR on the simulated datasets with 20% error rate. As can be seen from Supplementary Table S14, kngMap correctly mapped the maximum number of reads and bases to the reference genome. Importantly, kngMap achieved 100% aligned coverage, demonstrating that all mapped reads by kngMap are completely aligned. For the base sensitivity and precision in Supplementary Table S14, we can see that kngMap still performed the best, indicating that most bases are correctly mapped in the alignments generated by kngMap. Similar mapping results can be found in Supplementary Table S15 with simulated dataset with 25% error rate. Therefore, these results in Supplementary Tables S14 and S15 demonstrate that our kngMap is more robust to sequencing errors, and it can obtain better mapping results for a higher error rate up to ∼15%.

**FIGURE 5:**
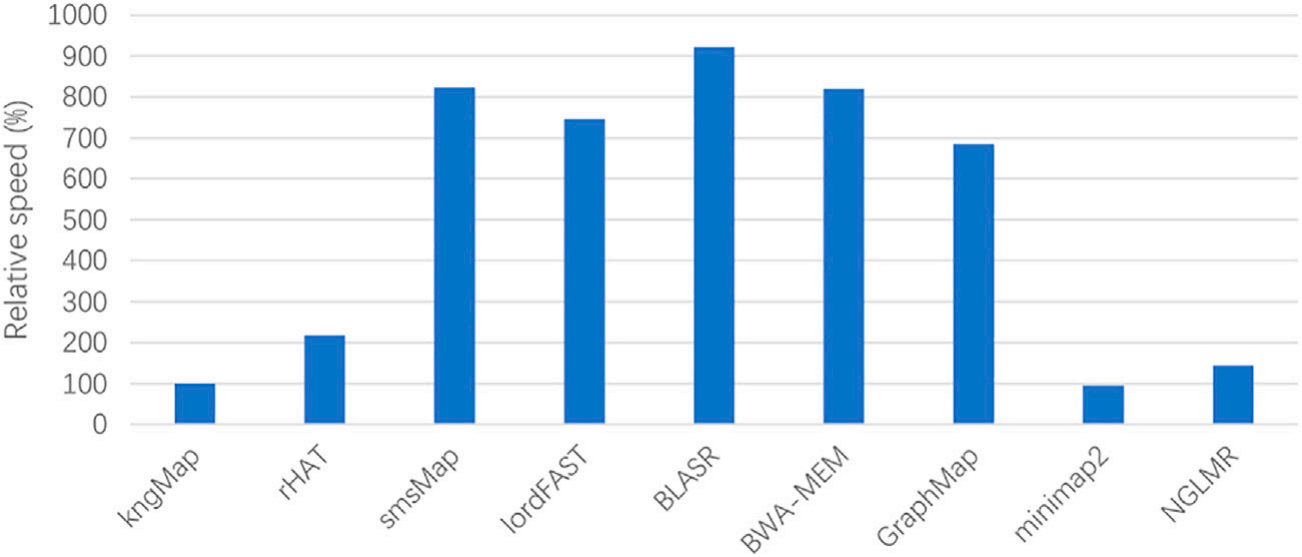
Relative speed of the aligners benchmarking on the real *H*. *sapiens* datasets. The relative speed of one mapper is defined as *T*(aln)/*T*(*kngMap*), where *T*(*aln*) and *T*(*kngMap*) are the alignment times of a compared aligner and kngMap, respectively. The lower the height of the bar, the faster the speed of the corresponding method. We can see that kngMap is faster than rHAT, smsMap, lordFAST, BLASR, BWA-MEM, GraphMap, and NGLMR mappers.

The massive amount of long noisy read data produced by SMS technologies brings some challenges to existing mapping approaches. In addition to accuracy, computational complexity is another important issue that needs to be considered. The time complexity of kngMap has four main components: 1) In the phase of constructing the reference genome index, it needs to extract all *k*-mers of the reference genome sequences to build the hash table index; thus, the maximum complexity is in the order of *O*(*G*), where *G* is the length of the genome sequences. 2) In the phase of generating *k*-mer *d*-neighborhood graph, it needs to retrieve all the matches of the *k*-mers for each query read, so the maximum complexity is *O*(N*L), where *N* is the number of reads, and *L* is the average read length. 3) In the phase of generating the skeleton of alignment, it needs to recurrently calculate the score of each node, so the maximum complexity is *O*(N*M), where *M* is the average number of matched *k*-mers for all reads. 4) In the phase of filling the gaps between anchors, it needs to obtain the detailed base-to-base alignment, so the maximum complexity is *O*(K*Q ^*2*^), where *K* is the average number of gaps, *Q* is the average length of gaps, and *Q<<L*. In summary, the total complexity for kngMap is *O*(G+N*L+N*M+K*Q ^*2*^). Since *Q<<L*, kngMap has a time complexity of the order of *G* and *L*. In order to graphically evaluate the computational efficiency of our kngMap, we compared kngMap with other mapping tools on the real *H*. *sapiens* datasets. Figure 5 shows the relative running time (wall-time) by using the nine tools. In terms of computational efficiency, we can see that the speed of kngMap is almost as fast as minimap2 and is several folds faster than other mapping methods. For example, kngMap is overall about 2-folds faster than rHAT and 6- to 9-folds faster than smsMap, lordFAST, BLASR, BWA-MEM, and GraphMap mappers. The speed comparison result described in Figure 5 indicates that kngMap is efficient to align SMS reads.

## 5 Conclusion

Since the infancy of SMS technologies (e.g., PacBio and Oxford Nanopore MinION) that produce longer but higher sequencing error reads, mapping these reads to a reference genome is often the most basic and computing-expensive step for downstream genome sequence analysis. Developing novel long read mapping tools is on demand for improving the mapping sensitivity and effectiveness of SMS read alignment.

In this study, we present kngMap, a new, fast, and highly sensitive mapping algorithm for long noisy reads. Mainly, kngMap contains three key characteristics: 1) kngMap proposes a scoring strategy in the chaining procedure to choose a group of anchors to form the initial alignment skeleton for each read. 2) kngMap designs an increased credibility function to refine the alignment skeleton. The scoring strategy and increased credibility function progressively ensure that kngMap can locate the aligned region for every query read, even in the situation that the matched seeds are dispersedly distributed in the reference genome. 3) For each of the gaps within the skeleton, kngMap classifies it into one of three categories and implements a specific alignment strategy to fill the corresponding gaps, which can help to robustly handle potentially different types of SVs in the reads. kngMap was benchmarked on simulated and real datasets across various genomes with other state-of-the-art mappers. The experimental results demonstrated that kngMap has higher accuracy and sensitivity that can correctly map more sequences and bases to the reference genome and achieves 100% aligned read coverage ratio; meanwhile, it also has good ability to span different types of SVs within the reads.

## Data Availability

The original contributions presented in the study are included in the article/Supplementary Material, further inquiries can be directed to the corresponding authors.
