# Supplementary material for: kngMap: Sensitive and Fast Mapping Algorithm for Noisy Long Reads Based on the K-Mer Neighborhood Graph
Source: Front Genet. 2022 May 5;13:890651. doi: 10.3389/fgene.2022.890651 (PMC9117619; doi:10.3389/fgene.2022.890651)
Supplement: Supplementary file 1 [file DataSheet1.docx]

**kngMap: sensitive and fast mapping algorithm for noisy long reads based on the *k*-mer neighborhood graph**

Ze-Gang Wei^1^, Xing-Guo Fan^1^, Hao Zhang^1^, Xiao-Dan Zhang_1_, Fei Liu^1^, Yu Qian*, Shao-Wu Zhang^2^

1 Institute of Physics and Optoelectronics Technology, Baoji University of Arts and Sciences, Baoji, 721016, China

2 Key Laboratory of Information Fusion Technology of Ministry of Education, School of Automation, Northwestern Polytechnical University, Xi’an, 710072, China

* Corresponding authors, Yu-Qian: [qianyu0272@163.com](mailto:qianyu0272@163.com), Shao-Wu Zhang: zhangsw@nwpu.edu.cn

**Supplementary file**

**Figures**

**Figure S1.** An example to show that two continuous *k*-mers in an error-free read are also matched in the continuous positions in the genome. For instance, *w_0_* and *w_1_* are two adjacent *k*-mers in an error-free read, their matched positions, *p_3_* and *p_4_*, are also adjacent in the genome sequence, that is, *pos*(*w_0_*) – *pos*(*w_1_*) = *p_4_* - *p_3_*, where *pos*(*w_0_*) and *pos*(*w_1_*) are the positions of *w_0_* and *w_1_* in the read. The dotted mapping positions formed the alignment region for the read.

**Figure S2.** An example to show that two continuous *k*-mers in a read with errors are not matched in the continuous positions in the genome. With the sequence errors caused by SMS technology, they can be matched with two discontinuous positions in the genome, for instance, *w_3_* and *w_4_* are two adjacent *k*-mers in the read, their matched positions, $p_{3}^{1}$ and $p_{4}^{1}$, are not adjacent in the genome sequence, that is, *pos*(*w_3_*) – *pos*(*w_4_*) ≠ $p_{4}^{1}$ - $p_{3}^{1}$, where *pos*(*w_3_*) and *pos*(*w_4_*) are the position of *w_3_* and *w_4_* in the read. The dotted mapping positions formed the alignment region for the read.


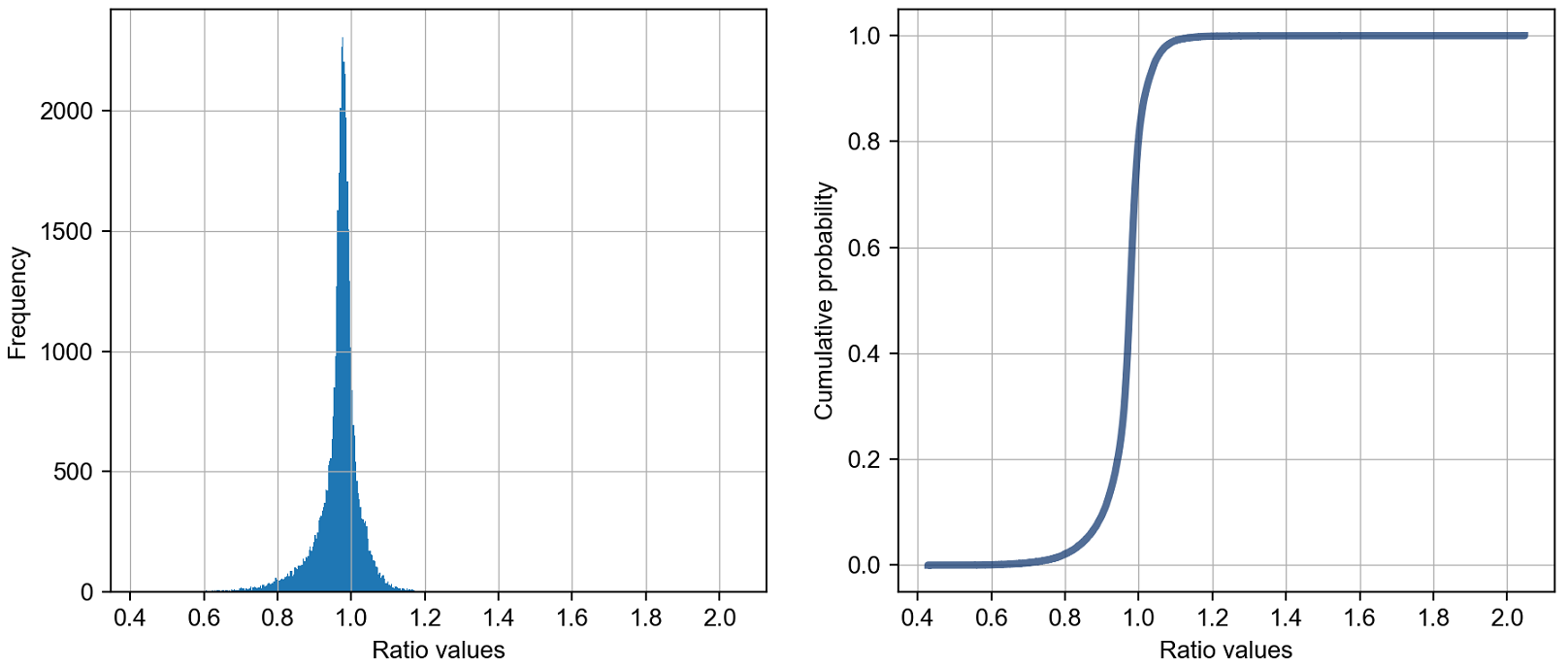


(a) (b)

**Figure S3.** The length ratio (defined as the length of aligned region in reference divided by the length of aligned region in read for one read) distribution of the aligned results. (a) Frequency distribution of the length ratio. (b) The cumulative distribution of the length ratio. From Figure (b) we can see that most values of the ratio are lower than 1.2, so we set $\lambda$ with 1.2. Here, the length ratio was calculated based on the alignment results on the real *C. elegans* (https://github.com/PacificBiosciences/DevNet/wiki/C.-elegans-data-set) dataset mapped by the BLASR mapping tool.

(a) (b)

**Figure S4.** An example to show the generating of the alignment skeleton by kngMap. (a) By setting to a constant value in the generating of initial alignment skeleton, two anchors with long distance can be successfully detected. (b) Otherwise, two chains will be generated.


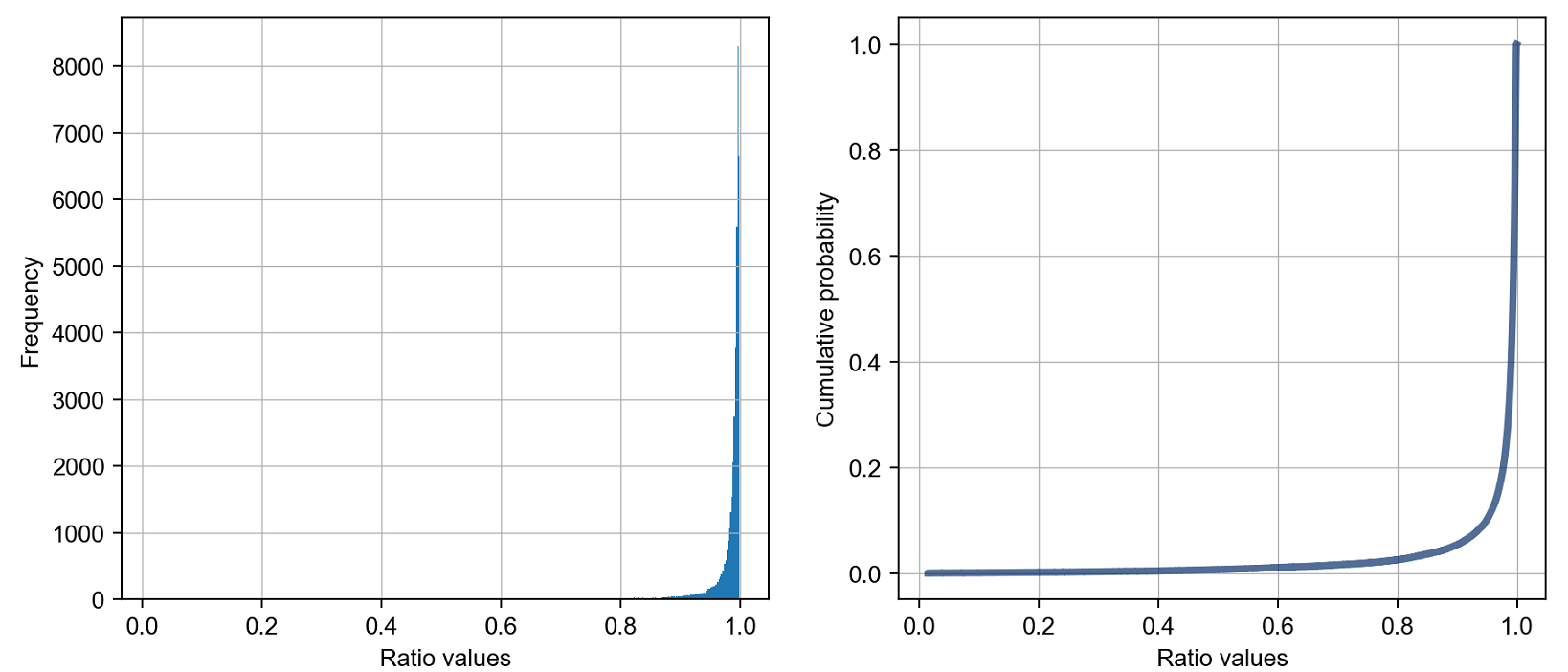


(a) (b)

**Figure S5**. The distribution of the mapped ratio, which is defined as the length of the region between the first matched *k*-mer and the last matched *k*-mer divided by the length of the mapped read. (a) Frequency distribution of the mapped ratio. (b) The cumulative distribution of the mapped ratio. From Figure (b) we can see that the maximum distance between two anchors for a read is usually smaller than the read length, indicating that the length of alignment skeleton is also smaller than the read length. Therefore, the fixed window length in the increased refining procedure is set with the read length. Similar to Figure S3, the mapped ratio values were calculated based on the aligned results obtained by BLASR mapping tool on the real *C. elegans* dataset.

1. (b)

**Figure S6**. A schematic illustration of the extension operation. (a) An illustration of the refined skeleton of alignment generated by kngMap. (b) An illustration of the partition of the read and local reference sequence by the refined skeleton of alignment. In this case, the skeleton (from *M_1_* to *M_7_*) partitions the read and the reference into 8 paired segments that need to be aligned. Each pair of unaligned segments, i.e., (*S_Ri_*, *S_Gi_*), i=1, 2, 3, 4, 5, 6, 7, will be aligned to compose the whole read alignment.


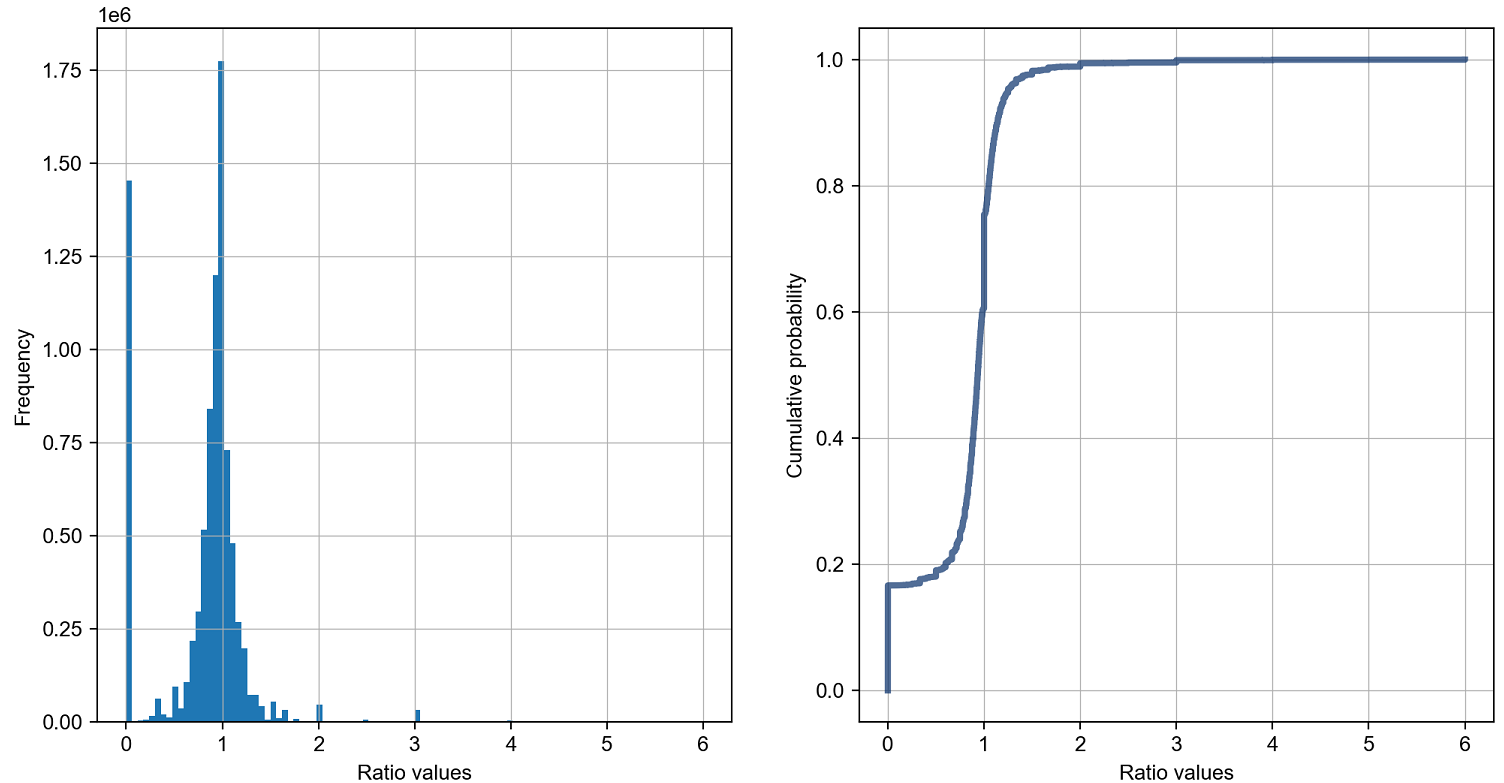


(a) (b)

**Figure S7**. The distribution of the adjacent *k*-mers length ratio, which is defined as the length of the gap in the reference divided by the length of the gap in the read anchored by adjacent *k*-mers. (a) Frequency distribution of the length ratio. (b) The cumulative distribution of the length ratio. From Figure (b) we can see that most values of the ratio are lower than 2, so that we set with 2. Similar to Figure S3, the aligned results were obtained by BLASR mapping tool on the real *C. elegans* dataset.

1. (b) (c)

**Figure S8**. A schematic illustration of filling match gaps. (a) For a match edge, if the two connected matches are corresponding to two neighboring seeding fragments on the read, LAMSA directly aligns the gapped part of the read (the yellow part between the blue and red blocks) against the corresponding local reference sequence.

**Figure S9**. The consecutiveness alignment of different methods on the real *H. sapiens* dataset.

**Supplementary Tables**

**Table S1**. Running command lines of different mapping programs.

| Programs | Running command lines |
| --- | --- |
| kngMap | locate genome.fa seq.fa >pos.txt |
|  | kngMap -g genome.fa -r seq.fa -p pos.txt -n seqNum -o aligned.sam |
| smsMap | locate --index genome.fa; locate -t 24 --search genome.fa --seq seq.fa >pos.txt |
|  | smsMap --seq seq.fa --genome genome.fa --pos pos.txt --out aligned.txt |
| BLASR | blasr sequences.fa genome.fa -m 0 --bestn 1 --nproc 24 > blasr_aligned.txt |
| minimap2 | minimap2 -t 24 -a genome.fa sequence.fa > minimap2.sam |
| GraphMap | graphmap align -r genome.fa -d sequence.fa -o graphmap_aligned.sam |
| NGMLR | ngmlr -t 24 -r genome.fa -q sequence.fa -o ngml.sam |
| S-conLSH | S-conLSH -a 1 --thread 24 ./ genome.fa sequence.fa > map.sam |
| conLSH | conLSH-indexer ./ ./genome.fa |
|  | conLSH-aligner ./ sequence.fa genome.fa > aligned.sam |
| lordFAST | lordfast --index genome.fa |
|  | lordfast -t 24 --search genome.fa --seq sequence.fa > map.sam |
| rHAT | rHAT-indexer ./ ref.fasta |
|  | rHAT-aligner -t 24 ./ reads.fa ref.fa > rhat.sam |
| BWA-MEM | bwa index genome.fa |
|  | bwa mem -t 24 -x pacbio genome.fasta sequence.fasta > bwa_mem.sam |

**Table S2**. Parameter settings of PBSIM2 software for generating simulated datasets.

| Ave. length (bp) | Command line |
| --- | --- |
| 10000 | pbsim --hmm_model P6C4.model --length-min 1000 --length-mean 10000 --depth 10 gen.fa |

**Table S3**. Read number and total bases of the simulated dataset (without structural variations).

| Error rates | Average length | Min. length | Max. length | Number of reads | Total bases |
| --- | --- | --- | --- | --- | --- |
| 15% | 10,157 | 1,000 | 71,616 | 100,000 | 1,015,730,844 |

**Table S4**. Different SVs types and its breakpoints in the genome.

|  | SV type | Start position | End position | Length |
| --- | --- | --- | --- | --- |
| 1 | inversion | 2553995 | 2554688 | 694 |
| 2 | inversion | 21205704 | 21205790 | 87 |
| 3 | deletion | 22565206 | 22565605 | 400 |
| 4 | deletion | 54823290 | 54823521 | 232 |
| 5 | insertion | 58690780 | - | 747 |
| 6 | deletion | 62084918 | 62089917 | 5000 |
| 7 | deletion | 62113214 | 62119658 | 6445 |
| 8 | deletion | 103514465 | 103526216 | 11752 |
| 9 | deletion | 105580458 | 105580969 | 512 |
| 10 | insertion | 143720173 | - | 133 |
| 11 | insertion | 160970493 | - | 62 |
| 12 | insertion | 192208930 | - | 503 |
| 13 | insertion | 228142855 | - | 210 |
| 14 | deletion | 229662172 | 229663217 | 1046 |
| 15 | inversion | 230458095 | 230458802 | 708 |
| 16 | deletion | 230458095 | 230459909 | 1815 |

**Table S5**. PacBio datasets website links

| Datasets | Website links |
| --- | --- |
| *E. coli* | <https://github.com/PacificBiosciences/DevNet/wiki/E.-coli-Bacterial-Assembly> |
| *A. thaliana* | <https://github.com/PacificBiosciences/DevNet/wiki/Arabidopsis-P5C3> |
| *H. sapiens* | https://github.com/PacificBiosciences/DevNet/wiki/H_sapiens_54x_release |

**Table S6**. Reference genome website links

| Genome | Website links |
| --- | --- |
| *E. coli UTI89* | https://www.ebi.ac.uk/ena/browser/view/ERX987748?show=reads |
| *A. thaliana* | <http://datasets.pacb.com.s3.amazonaws.com/2014/Arabidopsis/reads/polished_assembly.fasta> |
| *H. sapiens* | https://www.ncbi.nlm.nih.gov/assembly/GCF_000306695.2/ |

**Table S7**. Statistics of *E. coli*, *A. thaliana* and *H. sapiens* datasets

| Datasets | Reference genome length | Read number | Total bases | Average read length |
| --- | --- | --- | --- | --- |
| *E. coli* | 4,681,865 | 42,582 | | 106,765,908 | | --- | | 2,507 |
| *A. thaliana* | 130,857,836 | 22,837 | 187,687,752 | 8,218 |
| *H. sapiens* | 3,037,883,181 | 65,970 | 479,791,292 | 7,272 |

**Table S8**. The number of mapped reads, mapped bases, mapped matched bases and the alignment score of nine methods on the real *H. sapiens* dataset.

| Methods | Mapped reads | Mapped bases | Matched bases | Alignment score |
| --- | --- | --- | --- | --- |
| kngMap | 65970 | 479791292 | **438663286** | **365297544** |
| rHAT | 65908 | 431916393 | 392393158 | 272296221 |
| smsMap | 65970 | 479791292 | 421103035 | 336241351 |
| lordFAST | 65454 | 454392327 | 419909044 | 336457708 |
| BLASR | 65,011 | 439,695,280 | 399375111 | 301276499 |
| BEA-MEM | 65071 | 433759717 | 407713525 | 316908089 |
| GraphMap | 63463 | 459626158 | 422545919 | 356531261 |
| Minimap2 | 64411 | 443959690 | 414877640 | 331254090 |
| NGMLR | 60992 | 409452245 | 382306956 | 290878061 |

**Table S10**. The consecutive alignment for nine mapping methods at different covering thresholds.

| Methods | Covering thresholds | | | |
| --- | --- | --- | --- | --- |
|  | 80% | 85% | 90% | 95% |
| kngMap | 100 | 100 | 100 | 100 |
| rHAT | 88.88 | 88.54 | 88.01 | 86.95 |
| smsMap | 100 | 100 | 100 | 100 |
| lordFAST | 95.52 | 95.22 | 94.95 | 94.68 |
| BLASR | 89.90 | 88.66 | 87.17 | 84.92 |
| BWA-MEM | 88.16 | 86.90 | 85.38 | 83.50 |
| GraphMap | 99.91 | 99.88 | 99.84 | 99.77 |
| minimap2 | 89.72 | 88.48 | 87.03 | 85.10 |
| NGLMR | 88.56 | 87.04 | 85.26 | 82.96 |

**Table S12**. Agreement of different alignment methods for real *H. sapiens* dataset

|  | kngMap | rHAT | smsMap | lordFAST | BLASR | BWA-MEM | GraphMap | minimap2 | NGMLR |
| --- | --- | --- | --- | --- | --- | --- | --- | --- | --- |
| kngMap | - | 84.67 | 79.10 | 87.31 | 72.12 | 76.57 | 90.60 | 81.02 | 74.18 |
| rHAT | 92.01 | - | 82.26 | 88.21 | 78.49 | 82.71 | 90.61 | 87.11 | 79.49 |
| smsMap | 91.25 | 83.74 | - | 87.39 | 80.91 | 76.98 | 89.09 | 81.14 | 74.27 |
| lordFAST | 92.61 | 85.45 | 81.55 | - | 74.42 | 78.98 | 90.91 | 82.71 | 75.56 |
| BLASR | 92.83 | 87.69 | 92.24 | 90.10 | - | 86.41 | 91.64 | 91.15 | 82.25 |
| BWA-MEM | 93.64 | 88.12 | 87.05 | 91.41 | 83.85 | - | 92.23 |  | 84.09 |
| GraphMap | 94.54 | 87.38 | 81.20 | 90.01 | 74.86 | 79.23 | - | 83.69 | 77.09 |
| minimap2 | 94.56 | 88.94 | 86.86 | 91.05 | 84.24 |  | 92.70 | - | 84.36 |
| NGLMR | 97.15 | 91.50 | 91.04 | 93.67 | 89.04 | 92.48 | 95.81 | 96.83 | - |

**Table S13**. The performance of each pair methods on *H. sapiens* dataset for which their alignments do not agree.

|  | kngMap | rHAT | smsMap | lordFAST | BLASR | BWA-MEM | GraphMap | minimap2 | NGMLR |
| --- | --- | --- | --- | --- | --- | --- | --- | --- | --- |
| kngMap | - | 10048  (94.28) | 13787  (7.61) | 7851  (25.91) | 17433  (28.09) | 14552  (26.06) | 3693  (-1.72) | 10956  (26.12) | 12051  (47.15) |
| rHAT | 5266  (-34.86) | - | 11692  (-12.96) | 7275  (-7.92) | 13234  (5.80) | 10520  (-3.50) | 3692  (-34.68) | 6972  (-13.66) | 8586  (25.60) |
| smsMap | 5766  (-10.13) | 10664  (65.91) | - | 7798  (17.47) | 5043  (-21.17) | 14286  (16.94) | 4685  (-14.20) | 10880  (15.35) | 11992  (35.40) |
| lordFAST | 4831  (0.06) | 9480  (89.16) | 12072  (7.13) | - | 15936  (26.50) | 13203  (20.63) | 3597  (-4.94) | 10150  (22.95) | 11423  (41.15) |
| BLASR | 4656  (30.07) | 7957  (43.69) | 11632  (32.24) | 6077  (5.34) | - | 8176  (3.87) | 3162  (-26.26) | 4507  (-12.69) | 7397  (35.72) |
| BWA-MEM | 4135  (4.74) | 7697  (89.06) | 8422  (5.81) | 5420  (38.82) | 9788  (22.19) | - | 2811  (-1.81) | 3973  (16.40) | 6164  (49.89) |
| GraphMap | 3461  (3.72) | 7958  (97.97) | 11928  (9.33) | 5976  (34.02) | 15233  (25.01) | 12543  (30.06) | - | 9112  (27.96) | 11137  (44.04) |
| minimap2 | 3501  (-1.82) | 7094  (91.66) | 8458  (2.06) | 5638  (34.47) | 9505  (16.39) | 7179  (16.06) | 2517  (-6.63) | - | 6278  (52.22) |
| NGLMR | 1738  (-38.32) | 5172  (56.56) | 5459  (-9.46) | 3747  (17.31) | 6560  (-4.58) | 4479  (3.00) | 1627  (-33.06) | 1553  (-38.14) | - |

**Table S14**. Mapping results of different mapping tools on the simulated human dataset with error rate of 20%. This dataset contains 10,000 reads and 1.015 billion bases.

| Methods | CMR | CMB | Aligned coverage (%) | Sensitivity (%) | Precision (%) |
| --- | --- | --- | --- | --- | --- |
| kngMap | 99,102 | 1,006,608,373 | 100 | 98.92 | 98.91 |
| rHAT | 98,124 | 996,674,538 | 98.07 | 97.38 | 97.72 |
| smsMap | 98,235 | 997,801,998 | 98.21 | 96.07 | 96.07 |
| lordFAST | 99,003 | 1,005,602,801 | 98.99 | 98.73 | 98.85 |
| BLASR | 98,369 | 999,163,075 | 98.09 | 97.88 | 98.14 |
| BWA-MEM | 98,145 | 996,887,841 | 98.02 | 97.59 | 97.78 |
| GraphMap | 97,152 | 986,801,646 | 97.14 | 96.03 | 95.90 |
| Minimap2 | 98,456 | 1,000,046,760 | 98.34 | 98.22 | 98.35 |
| NGMLR | 96,267 | 977,812,439 | 96.10 | 93.83 | 94.98 |

**Table S15**. Mapping results of different mapping tools on the simulated human dataset with error rate of 25%. This dataset contains 10,000 reads and 1.015 billion bases.

| Methods | CMR | CMB | Aligned coverage (%) | Sensitivity (%) | Precision (%) |
| --- | --- | --- | --- | --- | --- |
| kngMap | 98,196 | 997,407,116 | 100 | 98.01 | 97.98 |
| rHAT | 96,257 | 977,712,094 | 96.20 | 95.53 | 95.86 |
| smsMap | 97,568 | 991,028,326 | 97.54 | 95.42 | 95.42 |
| lordFAST | 97,311 | 988,417,898 | 97.30 | 97.04 | 97.16 |
| BLASR | 90,282 | 917,022,172 | 90.02 | 89.83 | 90.07 |
| BWA-MEM | 96,987 | 985,126,929 | 96.87 | 96.44 | 96.62 |
| GraphMap | 97,254 | 987,838,931 | 97.24 | 96.13 | 96.03 |
| Minimap2 | 96,089 | 976,005,666 | 95.98 | 95.86 | 95.99 |
| NGMLR | 95,365 | 968,651,774 | 95.20 | 92.95 | 94.09 |
